# Evaluation of monotherapy of Coenzyme Q10, L-carnitine or combined therapy on semen parameters in idiopathic male infertility: A placebo-controlled double blind randomized clinical trial

**DOI:** 10.1080/20905998.2025.2509424

**Published:** 2025-05-24

**Authors:** Ahmed Higazy, Waleed Mohammed, M. Esmat, Mohamed Samir

**Affiliations:** Faculty of Medicine, Ain Shams University, Cairo, Egypt

**Keywords:** Coenzyme Q10, DNA fragmentation, L-carnitine, male infertility, oligoasthenozoospermia, oxidative stress

## Abstract

**Introduction:**

Antioxidant therapy has been proposed to improve the semen parameters in idiopathic male infertility with no clear consensus on the proper way of management. Our study aimed to evaluate the monotherapy of Coenzyme Q10 (CoQ10), L-Carnitine, or combined therapy on semen parameters in idiopathic male infertility presented with oligoasthenoteratozoospermia (OAT).

**Materials and methods:**

Two hundred patients presented with OAT were randomly allocated into four equal groups representing CoQ10, L-Carnitine, or combined therapy of both versus placebo. Our primary outcome was to monitor the improvement in semen parameters. The secondary outcomes were to evaluate the impact on the sperm DNA fragmentation (SDF) index and hormonal profile.

**Results:**

After 3 months of medical therapy, 174 patients completed the follow-up period. A highly statistically significant difference was monitored in the monotherapy and combination therapy in all semen parameters and SDF index compared to the baseline, while the placebo group failed to show any improvement. A rise in testosterone level and a decrease in luteinizing hormone were seen in the monotherapy and combination therapy, while no change was noted in the placebo group. Prolactin levels showed no change in all groups.

**Conclusion:**

Antioxidant therapy improves semen parameters in patients with OAT. A combination therapy of CoQ10 and L-carnitine results in a superior improvement in semen parameters compared to monotherapy in men with idiopathic OAT.

## Introduction

Male factors in infertility represent about 50% of cases. Multiple factors may be related to male infertility, such as genetic disorders, developmental disturbances, varicocele, endocrine abnormalities, immunological and genital tract infections, and environmental as well as systemic diseases. Idiopathic male infertility is found in about 30% of male infertility cases when the underlying etiology is unknown [1,2].

Oxidative stress (OS) induced by reactive oxygen species (ROS) is one of the known causes of idiopathic male infertility. ROS damages the sperm plasma membrane via DNA fragmentation and lipid peroxidation. This decreases sperm mobility and vitality, thereby reducing sperm fertilizing capacity. Sperm DNA fragmentation (SDF) is an uncorrectable step that causes male infertility, and several laboratory tests are used to identify it [3–5].

L-carnitine is an antioxidant that prevents ROS from damaging sperm mitochondria. It enhances the antioxidative properties of the spermatozoa by acting as a free-radical scavenger and improving sperm fertilization capacities [6].

Coenzyme Q10 (CoQ10) is another antioxidant in the seminal respiratory chain. It is crucial for many semen parameters, such as motility and count. Therefore, several studies have been conducted and found that CoQ10 supplementation improved pregnancy rates in infertile men [7–9].

Despite this, there is no clear consensus regarding the role of antioxidant therapy in male infertility with regards to specific antioxidant therapy to use or the therapeutic dose and regime to improve semen parameters and enhance pregnancy.

Throughout our study, we aimed to evaluate the role of L-carnitine, CoQ10, and their combination in treating idiopathic male infertility.

## Materials and methods

Between August 2023 and October 2024, two hundred male patients with idiopathic primary infertility were enrolled in our study who were presented with oligoasthenoteratozoospermia (OAT) or affection in two parameters (motility, count, morphology) below normal levels based on two semen analyses that were done 1 month apart [10]. A detailed history was obtained from all patients in addition to a generalized and focused genital examination to rule out any possible cause of infertility and ensure 12 months of regular unprotected intercourse. Patients with azoospermia, cryptorchidism, varicocele, endocrine disorder, systemic disease, relevant medication history that may impact spermatogenesis, female factor, previous antioxidant medication, genital infection, testicular malignancy, or scrotal surgeries were excluded from our study. Varicocele was evaluated using both clinical examination and testicular Doppler. Urinary tract and male accessory gland infections were evaluated and excluded from our study.

Smoking history and body mass index (BMI) were evaluated in our study population. The smoking index was calculated into three levels: mild smoking (smoking index ≤ 200), moderate smoking (200 < smoking index < 400), and severe smoking (smoking index ≤ 400) [11].

OAT was defined based on the 6^th^ edition of the WHO criteria for semen analysis 2021 using Computer-assisted semen analysis (CASA) by Intelligent CASA analyzer of Delta care provider, Cairo, Egypt, manufactured in China [10]. Semen analysis was evaluated using an automated semen analyzer. After 3–5 days of sexual abstinence, ejaculates were collected at the laboratories and were kept at 37 °C before analysis. Patients were informed about the importance of reporting any missed ejaculate fractions. Ejaculates were collected at the laboratories. And specimens were kept at 37°C before initiation of and during the analysis in case of sperm motility assessment.

TUNEL Assay (Terminal deoxynucleotidyl transferase dUTP nick end labeling) with Fluorescence Microscopy (Olympus BX51) labels broken DNA ends in sperm with fluorescent nucleotides, allowing for quantification of DNA fragmentation. to evaluate SDF in our study [12]. SDF was evaluated simultaneously with the semen analysis after 3 days of abstinence, and data were recorded. SDF index of 25% or more was considered significant in our study [13].

After fulfilling the inclusion criteria, informed consent was obtained from all patients. Patients were randomly allocated into 4 equal groups using sealed envelopes for concealed random allocation with equal ratios that were prepared by a third party not involved in the study.

Different groups were scheduled for 3 months of therapy. Group 1 received a daily dose of 200 mg of CoQ10, group 2 received L-carnitine 1 gm per day, group 3 received a combination of 200 mg of CoQ10 and 1 gm of L-carnitine per day, and group 4 received a placebo for the same schedule. Treatment allocation was concealed from patients, the data collectors, and the statistician.

Patients were followed up after 3 months of treatment by semen testing, and the results were compared to the baseline results before treatment and with other groups. All semen evaluations in the follow-up were done in the same lab by the same investigator.

Our primary outcome was to evaluate the change in semen parameters in response to antioxidant medications. Our secondary outcome was to evaluate the response of the SDF index and change in hormonal profile, including serum testosterone, follicle-stimulating hormone (FSH) and luteinizing hormone (LH) levels, and prolactin, in response to medication. Hormonal profile was measured using ROCHE Cobas e411 device and blood testing was done between 7 and 11 AM.

### Statistical analysis

Study data were analyzed by the Statistical Package for Social Science (IBM SPSS) version 23. The comparison between groups regarding qualitative data was done by using Chi-square test. The comparison between two independent groups with quantitative data and parametric distribution was done by using an independent t-test while between two paired groups with quantitative data and parametric distribution was done by using a Paired t-test. The confidence interval was set to 95% and the margin of error accepted was set to 5%. A p-value <0.05 was considered significant and a P-value <0.01 was considered highly significant.

## Results

**Figure 1.**
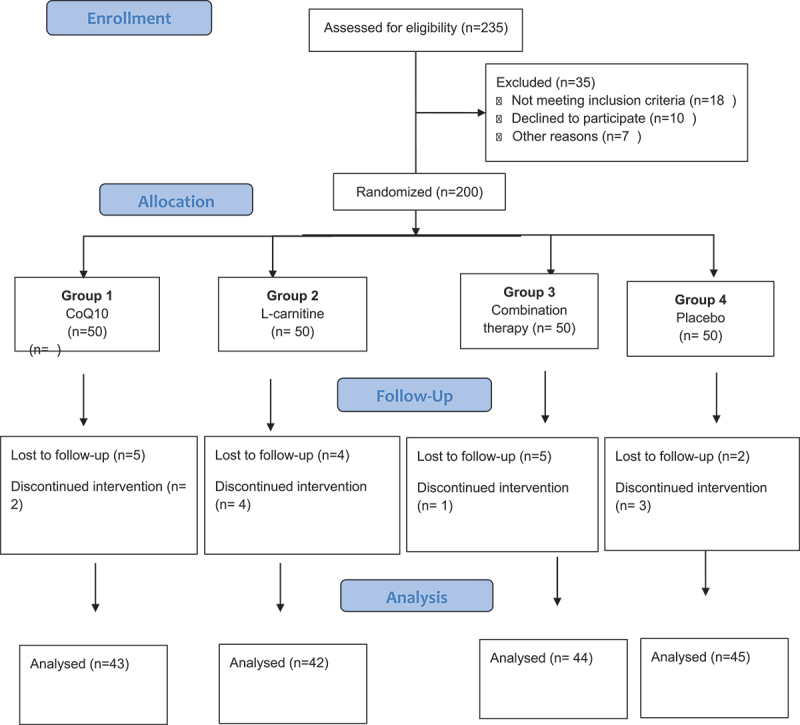
Consort flow chart.

Out of 200 patients who were initially included in our study, 174 patients were compliant to the medication and completed the 3-month follow-up period and were included in our final evaluation as shown in (Figure 1).

Patients’ characteristics were demonstrated in (Table 1) with no statistically significant difference between our study groups in terms of age, BMI, and smoking history as potential factors influencing male fertility.

**Table 1.** Patients characteristics.

	Group 1 (CoQ10) N = 43	Group 2 (L-carnitine) N = 42	Group 3 (Combination) N = 44	Group 4 (Placebo) N = 45
Age	27.47 ± 2.91	27.12 ± 4.80	28.14 ± 5.47	27.18 ± 5.62
BMI	26.83 ± 4.46	27 ± 3.41	26.63 ± 4.26	27.83 ± 4.56
Number of smoker and Smoking Index	12 Mild (5) Moderate (3) Severe (4)	11 Mild (7) Moderate (1) Severe (3)	16 Mild (5) Moderate (10) Severe (1)	14 Mild (4) Moderate (9) Severe (1)

Change in semen parameters following 3 months of medication was reported in (Table 2). A highly statistically significant difference was seen in groups 1,2 and 3 compared to their baseline level regarding all semen parameters (count, motility, morphology, and SDF index) while group 4 representing placebo showed no change in the semen parameters. Improvement in semen parameters was seen higher in group 3 representing the combination therapy in comparison to the monotherapy of groups 1 and 2 in all parameters as shown in (Table 2).

**Table 2.** Semen parameters.

Semen parameters		Group 1 (CoQ10) N = 43	Group 2 (L-carnitine) N = 42	Group 3 (Combination) N = 44	Group 4 (Placebo) N = 45	P-value
Semen volume (ml)	Before treatment	2.69 ± 0.65	2.48 ± 0.47	2.66 ± 0.59	2.37 ± 0.39	0.180
	After treatment	4.72 ± 0.75	4.61 ± 0.73	4.90 ± 1.04	4.44 ± 0.46	0.282
	P-value	0.324	0.845	0.824	0.106	
	CI (95%)	[4.496–4.944]	[4.389–4.831]	[4.593–5.207]	[4.306–4.574]	
Sperm concentration (million/ml)	Before treatment	13.42 ± 2.95	13.26 ± 2.92	12.93 ± 2.51	13.24 ± 2.63	0.657
	After treatment	26.86 ± 7.86	20.24 ± 4.32	32.93 ± 2.51	13.80 ± 2.50	0.001
	P-value CI (95%)	0.001 [24.511–29.209]	0.012 [18.934–21.546]	0.001 [32.188–33.672]	0.133 [13.070–14.530]	
Progressive motility (%)	Before treatment	23.26 ± 6.07	22.64 ± 6.30	23.25 ± 6.00	24.56 ± 5.40	0.268
	After treatment	34.91 ± 5.74	29.43 ± 5.13	38.52 ± 6.34	25.84 ± 5.69	0.001
	P-value	0.001	0.025	0.001	0.488	
	CI (95%)	[33.194–36.626]	[27.879–30.981]	[36.647–40.393]	[24.178–27.502]	
Total motility (%)	Before treatment	38.19 ± 6.73	37.19 ± 5.31	38.25 ± 6.67	38.82 ± 7.68	0.127
	After treatment	45.63 ± 6.65	40.26 ± 4.60	50.16 ± 2.92	39.16 ± 8.61	0.001
	P-value	0.001	0.026	0.001	0.184	
	CI (95%)	[43.619–47.641]	[38.869–41.651]	49.297–51.023]	[36.644–41.676]	
Morphology index (%)	Before treatment	18.77 ± 4.13	19.33 ± 4.25	18.75 ± 4.13	19.18 ± 4.22	0.106
	After treatment	25.53 ± 5.87	22.45 ± 4.94	29.89 ± 7.47	20.08 ± 4.22	0.020
	P-value	0.001	0.013	0.001	0.092	
	CI (95%)	[23.776–27.284]	[21.092–23.808]	[27.683–32.097]	[18.847–21.313]	
Sperm DNA fragmentation index	Before treatment	31.19 ± 4.64	29.62 ± 5.35	30.39 ± 2.09	30.80 ± 4.84	0.607
	After treatment	19.19 ± 4.64	23.62 ± 5.35	13.93 ± 3.23	29.56 ± 4.65	0.001
	P-value	0.001	0.028	0.001	0.268	
	CI (95%)	[17.803–20.577]	[22.002–25.238]	[12.976–14.884]	[28.201–30.919]	

Hormonal profile change was reported in (Table 3). A rise in testosterone level and a drop in LH level was seen in groups 1,2 and 3 while no change was seen in group 4. Prolactin levels showed no change in all groups.

**Table 3.** Hormonal profile.

Hormonal profile		Group 1 (CoQ10) N = 43	Group 2 (L-carnitine) N = 42	Group 3 (Combination) N = 44	Group 4 (Placebo) N = 45	P-value
Testosterone	Before treatment	3.98 ± 0.91	4.57 ± 1.01	3.87 ± 0.97	3.86 ± 0.81	0.261
	After treatment	5.73 ± 1.32	4.97 ± 1.09	6.51 ± 1.63	3.93 ± 0.83	0.001
	P-value	0.001	0.017	0.001	0.089	
	CI (95%)	[5.335–6.125]	[4.640–5.300]	[6.028–6.992]	[3.687–4.173]	
FSH	Before treatment	8.50 ± 1.96	8.30 ± 1.83	8.56 ± 2.14	8.65 ± 1.82	0.244
	After treatment	6.41 ± 1.48	7.14 ± 1.57	5.17 ± 1.29	8.22 ± 1.73	0.001
	P-value	0.001	0.018	0.001	0.163	
	CI (95%)	[5.968–6.852]	[6.665–7.615]	[4.789–5.551]	[7.715–8.725]	
LH	Before treatment	8.07 ± 2.21	8.35 ± 2.17	8.12 ± 2.21	8.09 ± 2.07	0.286
	After treatment	5.24 ± 1.21	6.95 ± 1.53	4.45 ± 1.11	7.86 ± 1.65	0.002
	P-value	0.001	0.017	0.001	0.203	
	CI (95%)	[4.878–5.602]	[6.487–7.413]	[4.122–4.778]	[7.378–8.342]	
Prolactin	Before treatment	10.11 ± 2.32	10.11 ± 2.22	10.01 ± 2.50	9.64 ± 2.02	0.954
	After treatment	10.11 ± 2.32	10.12 ± 2.23	10.00 ± 2.50	9.67 ± 2.03	0.348
	P-value	0.324	0.778	0.4	0.481	
	CI (95%)	[9.417–10.803]	[9.446–10.794]	[9.261–10.739]	[9.077–10.263]	

## Discussion

One of the main explanations for idiopathic male infertility is the ROS, which represents a state of imbalance between pro-oxidants and antioxidants in seminal fluid. Although ROS is required for certain physiological processes during fertilization, the excess ROS results in damage to the sperm plasma membrane and sperm DNA fragmentation. This sperm insult affects the count, motility, and vitality, eventually undermining a successful fertilization [4,13].

L-carnitine as an antioxidant has been shown to provide energy for spermatozoa and protective function against ROS. CoQ10 is an antioxidant known also as ubiquinone which is a respiratory chain component. Its presence in the seminal fluid acts as an antioxidant in addition to mitochondrial bioenergetics and metabolic processes. Supplementation with CoQ10 has been shown to improve semen parameters and its seminal concentration correlates with such improvement [9,14,15].

Antioxidant therapy has been suggested as a potential therapy to counteract the hazardous effect of ROS. It was supported by 2 Cochrane systematic reviews and meta-analyses that it has a positive impact on pregnancy rate and live birth in sub-fertile couples [16,17]. On the other hand, the Males, Antioxidants, and Infertility (MOXI) trial reported that antioxidant therapy did not improve semen parameters or DNA integrity compared to placebo [18].

For this conflicting evidence, no clear recommendation can be drawn regarding the usage of antioxidant therapy in sub-fertile men to improve semen parameters and pregnancy rate.

Age, smoking, environmental factors like pollution, or unhealthy diet Smoking have been recognized as risk factors for oxidative stress that may impair fertility. In our study, Patients’ characteristics including age, BMI, and smoking showed no statistically significant difference between groups.

Our study showed improvement in semen parameters (increase of semen volume, sperm concentration, progressive motility, and reduction in abnormal forms) which was statistically significant in patients receiving L-carnitine, coQ10, and the combination therapy after 3 months compared to their baseline parameters, while no statistically significant difference was noted in the placebo group compared to the baseline parameters. Improvement seen in our study was higher in patients who received combination therapy than monotherapy of L-carnitine or coQ10 as shown in (Table 2).

In line with our findings, Kopets et al. found that the combination of 1990 mg of L-carnitine and L-acetyl-carnitine with coQ10, selenium, glutathione, folic acid, zinc, cyanocobalamin, and L-arginine showed more statistically significant improvement of semen parameters and pregnancy rate in infertile males than the placebo arm after 4 months of treatment without reported side effects [19].

Elnashar et al. demonstrated in their cross-over placebo-controlled study that the combination of 300 mg of L-carnitine Tartrate with vitamin B 6, vitamin E, vitamin B 12, vitamin D, vitamin C, zinc, selenium, folic acid, and CoQ 10 is effective and safe in males with idiopathic infertility [20].

In contrast, Steiner et al in their multicenter trial evaluated the combination of antioxidants (400 mg of vitamin E, 500 mg of vitamin C, 1 gm L-carnitine, 20 mg of zinc, 1000mcg folic acid, 10 mg of lycopene, and 0.20 mg selenium) in comparison to the placebo, failed to show any improvement of the semen parameters. They explained their findings by the fact that the study population was unlikely to get benefit from treatment with antioxidants and they were to include only male patients with high ROS levels [18], Ma et al. evaluated L-carnitine versus CoQ10 and vitamin E. L-carnitine improved all semen parameters and showed superiority to the combination therapy of CoQ10 and vitamin E [21].

SDF has been proposed as a marker of defective spermatogenesis in response to reactive oxygen species and oxidative stress leading to DNA fragmentation and eventually impairs sperm function. Although its role is not yet confirmed, nor its cut-off value or the appropriate way of measurement, many studies have reported its impact on fertility and successful pregnancy [22].

A threshold of SDF index of 25%, as measured by SCSA, is associated with reduced pregnancy rates by natural conception or intrauterine insemination, while a threshold value > 50% is associated with a poor outcome by in vitro fertilization [8].

In our study, improvement in the SDF was noticed in both monotherapy and combination therapy compared to the baseline parameters and Placebo. This improvement was highly significant in the combination group and the CoQ10 group with a more significant improvement in the combination group. In line with our results, Alahmar et al. reported a considerable improvement in SDF using CoQ10 in their case-control study [9].

Antioxidants reduce the oxidative stress in the testicular tissue promoting cellular function including Leydig cell function. L-carnitine has been shown to enhance mitochondrial beta-oxidation, leading to increased energy availability for testosterone synthesis in Leydig cells. CoQ10 supplementation improves mitochondrial function in testicular cells promoting Leydig cell function and potentially influencing testosterone biosynthesis rather than a direct effect on the hypothalamic-pituitary-gonadal axis [23].

Regarding post-treatment hormonal levels, our results showed increased plasma testosterone levels alongside decreased FSH and LH levels, indicating a positive effect on hormonal balance related to spermatogenesis. Importantly, no significant changes were observed in prolactin levels post-treatment, suggesting that while the combination therapy positively influences various aspects of male fertility, it does not adversely affect hormonal regulation associated with reproductive health.

Ma et al, reported a significant increase in testosterone and LH levels with L-carnitine therapy while CoQ10 showed an increase in testosterone levels only [21]. while Elnashar et al. reported no considerable change in the hormonal profile [20].

Despite the considerable improvement in the semen parameters noted in our study. the Males, Antioxidants, and Infertility (MOXI) randomized controlled trial reported no benefit from antioxidant intake on semen parameters on 383 participants [18]. Even in studies that reported improvement, the reported data showed wide heterogeneity in the rate of improvement and the recommended duration and dosage of medication. Some studies reported concerns about the hazardous effect of high doses of antioxidants with a paradoxical effect [1925]. This data heterogenicity makes it difficult to draft a clear recommendation for antioxidant usage.

According to our knowledge, no previous study has evaluated L-carnitine, CoQ10, or their combination in a placebo-controlled randomized controlled trial. Despite promising findings, several limitations exist in our study. It was a single-site study with a relatively small number of participants. We did not evaluate the pregnancy rate, as it is difficult to follow up with patients after conception where some participants will drop out, and, in our clinic, we evaluate male patients only and we can’t trace the pregnancy rate. The study evaluated treatment for 3 months with a noted improvement while in actual life, whether the patient should be treated till conception or not, this is not addressed in our study. We did not evaluate the serum or seminal level of L-carnitine or CoQ10 and correlate it with the change in semen parameters. Finally, we could not measure the change of ROS in the seminal fluid in response to treatment.

## Conclusion

Combination therapy of L-carnitine and CoQ10 showed a superior improvement in semen parameters and SDF index compared to monotherapy in patients with OAT.

## Data Availability

The datasets used and/or analyzed during the current study are available from the corresponding author upon reasonable request without breach of any confidential information regarding the study population.

## Sample size calculation

It was done by a separate member not involved in the study through the community department, Faculty of Medicine, Ain Shams University, by using the power analysis and sample size software (PASS 15) (version 15.0.10) for sample size calculation, and setting power at 80%, alpha error 0.05 and considering 10% dropout rate, a sample size of at least 30 patient in each group was considered sufficient to achieve study objective.
